# Mosaic crack mapping of footings by convolutional neural networks

**DOI:** 10.1038/s41598-024-58432-w

**Published:** 2024-04-03

**Authors:** Apichat Buatik, Phromphat Thansirichaisree, Phisutwat Kalpiyapun, Navid Khademi, Ittipon Pasityothin, Nakhorn Poovarodom

**Affiliations:** 1 Research Unit of Infrastructure Inspection, Monitoring, Repair and Strengthening, Faculty of Engineering, Thammasat School of Engineering, Thammasat University, Pathumthani, Thailand; 2 School of Civil Engineering, College of Engineering, University of Tehran, Tehran, Iran

**Keywords:** Civil engineering, Imaging techniques, Information technology

## Abstract

Cracks are the primary indicator informing the structural health of concrete structures. Frequent inspection is essential for maintenance, and automatic crack inspection offers a significant advantage, given its efficiency and accuracy. Previously, image-based crack detection systems have been utilized for individual images, yet these systems are not effective for large inspection areas. This paper thereby proposes an image-based crack detection system using a Deep Convolution Neural Network (DCNN) to identify cracks in mosaic images composed from UAV photos of concrete footings. UAV images are transformed into 3D footing models, from which the composite images are created. The CNN model is trained on 224 $\times$ 224 pixel patches, and training samples are augmented by various image transformation techniques. The proposed method is applied to localize cracks on composite images through the sliding window technique. The proposed VGG16 CNN detection system, with 95% detection accuracy, indicates superior performance to feature-based detection systems.

## Introduction

Monitoring structural health and the dependability of infrastructure requires regular condition assessments. Surface cracks are a significant sign of structural deterioration, which, if ignored, could lead to severe damage. To prevent further deterioration of building damage and, ultimately, the collapse of structures, robust crack detection has thus shown to be crucial for structural health monitoring. Visual inspection has always been the standard procedure to find and assess flaws in concrete structures. This inspection method’s drawbacks have been recognized, including its expense, time commitment, labor-intensive manual inspection, proneness to error, and restriction on inspection frequency. Therefore, interesting alternatives to human crack inspection are image-based automation approaches.

Typically, image-based crack detection algorithms extract crack pixels using pre-processing techniques^1^. Several researchers have suggested modifications to improve the method’s efficacy and accuracy. Fujita et al.^2^ suggested removing noise from pictures using two pre-processing methods. The technique uses a subtraction procedure to eliminate shading and erroneous lighting. Throughout this process, the utilization of line filters based on the Hessian matrix enhances the line features associated with cracks. In the second step, the thresholding procedure is used to extract fracture sections once the noise has been removed. Tong et al.^3^ have proposed a method for identifying cracks at the surface of concrete bridges’ bottom. Their suggested method eliminates image noise by using a morphological procedure to separate images into crack and non-crack. Yamaguchi and Hashimoto^4^ used a technique based on the percolation model to find cracks in the pavement’s support structures. The technique starts by setting up a seed crack pixel, after which the percolation process labels the nearby pixels. Abdel-Qader et al.^5^ evaluated several edge detection methods, including the Fast Fourier Transform (FFT), Fast Haar Transform (FHT), and Sobel edge detector for crack pixel extraction. Based on the 86% accuracy score, the results show that the FHT technique has a superior ability to remove noise from textured surface clutter.

Several researchers have recently used machine learning strategies, including Support Vector Machines (SVM)^6,7^ and Neural Networks^8^ to identify features from infrastructure images and categorize them as crack or non-crack. Liu et al.^9^ utilized picture intensity attributes and the Support Vector Machine (SVM) to locate tunnel cracks. Prasanna et al.^10,11^ suggested a histogram-based crack detection system for a concrete bridge deck that included the Spatially Tuned Robust Multi-feature (STRUM) classifier for automatic crack identification. The result of these investigations is a system combining intensity-based, gradient-based, and scale-space features into a single feature vector for use as input by various machine learning classifiers, including SVM, Adaboost, and Random Forest. This unified feature vector demonstrates the STRUM classifier’s superiority over previous image-based crack detection techniques. However, image noise resulting from distortion and lighting fluctuations on the real structures negatively impacted the method’s outcomes. For crack inspection, the noise continues to be a big challenge. Digital camera or unmanned aerial vehicle (UAV) photos, for instance, may have shading, dust specks, or black stains. These Backscatter marks make it difficult for image-based methods to distinguish between cracks and non-cracks.

Given its versatility, the Convolutional Neural Network (CNN) is used as a feature-learning tool to overcome the challenges posed by image processing techniques^12^. The CNN^13^ is typically employed for its image recognition and classifying photos into various groups^14^. However, researchers such as Zhang et al.^15^ have used low-cost smartphone photos and deep convolutional neural networks to detect road cracks. Cha et al.^12^ focused on the viability of using Deep Convolutional Neural Networks (DCNN) to detect concrete cracks automatically, and they achieved a 98% accuracy rate. The outcomes of their investigation, which relied on 40,000 (256 $\times$ 256 pixel) training images, show that DCNNs perform better than conventional techniques. Despite the advantages of the automated CNN system, one must consider the enormous amount of necessary training data, which adds to the system’s high computational cost. For these reasons, parallel graphics processing units (GPUs) and pre-trained networks are favored for training. Pre-trained networks such as the AlexNet^14^, VGG16^16^, and ResNet^17^ models are popular and reliable small-scale datasets^15^. Gopalakrishnan et al.^18^ have suggested using pre-trained deep-learning models on UAVs to detect cracks in civil infrastructure. The proposed method produced 90% accurate outcomes. Nevertheless, they did not use any pre-processing or data augmentation techniques. Without data augmentation, overfitting could have another negative effect. An effective way to lessen overfitting is still to augment data^14^. Ozgenel and Sorguc^19^ examined the AlexNet^14^, VGG16 and VGG19^16^, GoogleNet^20^, ResNet101, and ResNet152^17^ pre-trained their models for their effectiveness in finding cracks in concrete constructions. The researchers discussed the quantity of the training data, the number of layers, and the learning parameters in the CNN when using these pre-trained networks. They concluded that, compared to other pre-trained networks, the VGG16 network^16^ exhibits preferred performance in masonry photos. Furthermore, despite the quantity of training data, ResNet findings demonstrated overfitting.

Note that RGB imaging is used in the approaches stated above. Mosaicking has, however, been included in a few studies^21–23^. A mosaic image that closely resembles the model of the real structure is produced by combining a number of various images with potential lighting adjustments. Yet, with the incorporation of a blending algorithm^24,25^, collated mosaic images could have blurry portions, making fracture detection techniques challenging to use. Prasanna et al.^11^ used a crack density map and image mosaic to show cracks on a bridge deck.

Conventional image-based crack detection systems may not be effective enough for large inspection areas, as they rely on individual images and cannot capture the entire structure. To address this limitation, this paper proposes a novel crack detection system using a CNN (the VGG16 network) to identify cracks in mosaic images composed from UAV photos of concrete footings. The proposed system utilizes 3D models to create high-quality mosaic images that accurately represent the entire structure. These mosaic images are then inputted to the CNN and trained to identify cracks based on their unique characteristics. This approach overcomes the limitations of conventional methods by enabling the detection of cracks in large areas with high accuracy. The proposed system demonstrates promising performance. This performance is significantly higher than that of other detection systems typically used for crack detection on individual images. The proposed system also offers several advantages over conventional methods, including its ability to handle large inspection areas and its ability to provide real-time results.

## Proposed methodology

**Figure 1:**
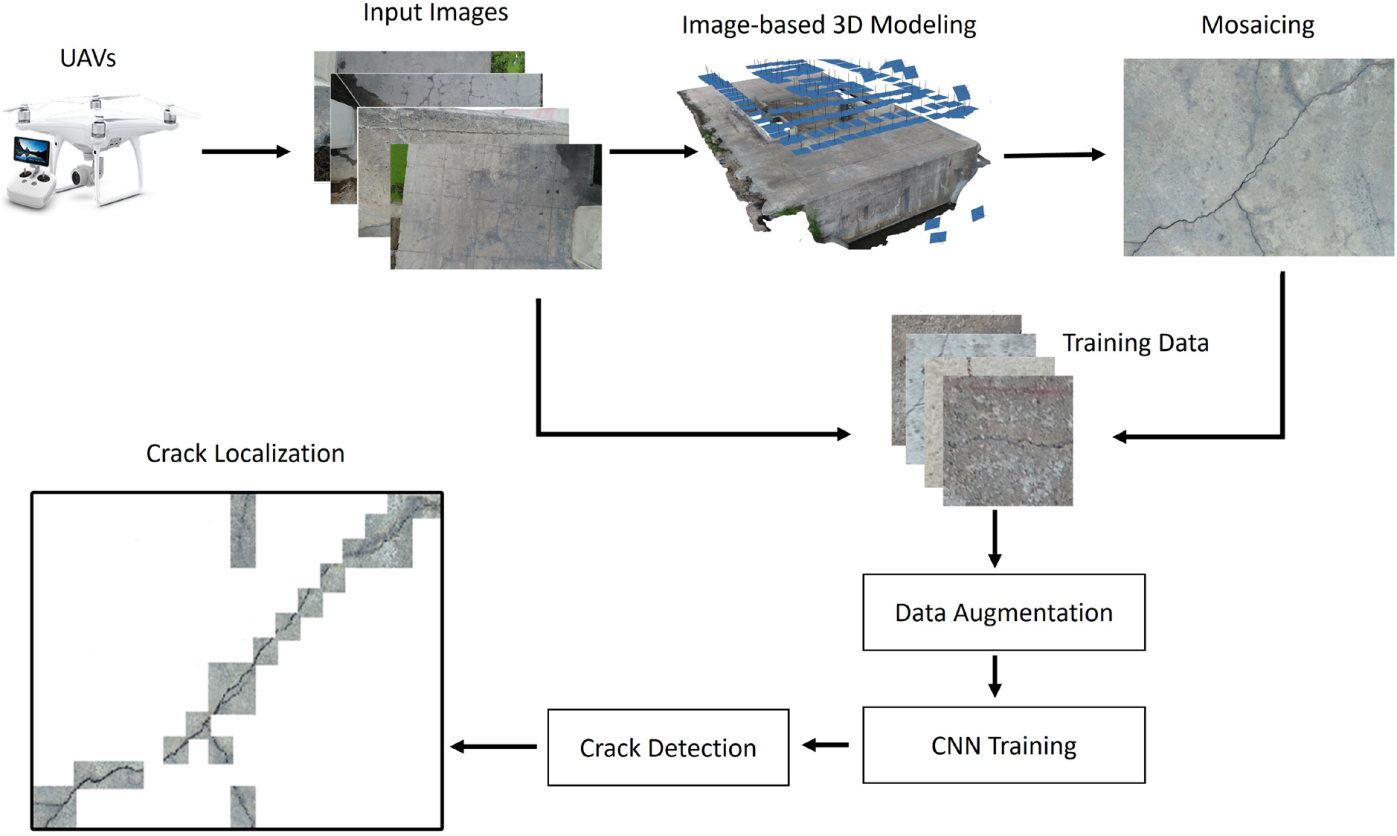
Proposed methodology.

Figure 1 lays out the approach for the suggested crack mapping system. CNN training encapsulates images acquired by using a UAV and a digital camera. The mosaic image is thereafter created from these input images to form a large model. A patch size of 224 $\times$ 224 is obtained from both RGB and mosaic images. Subsequently, the patch size is labeled manually to create a dataset to train the CNN. The crack locations are presented in a binary map and then blended with an RGB image, and the mosaic is used to assess how well the proposed system performed after the model training. Each module represented in Fig. 1 is described hereafter.

### Image procurement

Unmanned aerial vehicles (UAVs) have recently become a practical alternative to conventional surveying techniques thanks to their distinct benefits in terms of speed, accessibility, and cost-effectiveness. In this line, the DJI Phantom 4 drones were used to take pictures close to the surface of concrete buildings. To obtain a detailed and comprehensive 3D model of a concrete building, Images of the whole surface of the structure must be taken. Achieving complete coverage of the building can be challenging, particularly when relying on manual data collection using drones. This problem can be solved by using a pre-planned flight route approach called a “sweeping strategy”. In the sweeping technique, the building’s surface is divided into a grid of cells, and the UAV is flown over each cell in a predetermined pattern while taking pictures of the surface. The photographs taken will always be of consistent quality and resolution because the UAV may be configured to fly at a constant height and speed. The building’s complete surface can be scanned by repeating this procedure for each grid cell.

**Figure 2:**
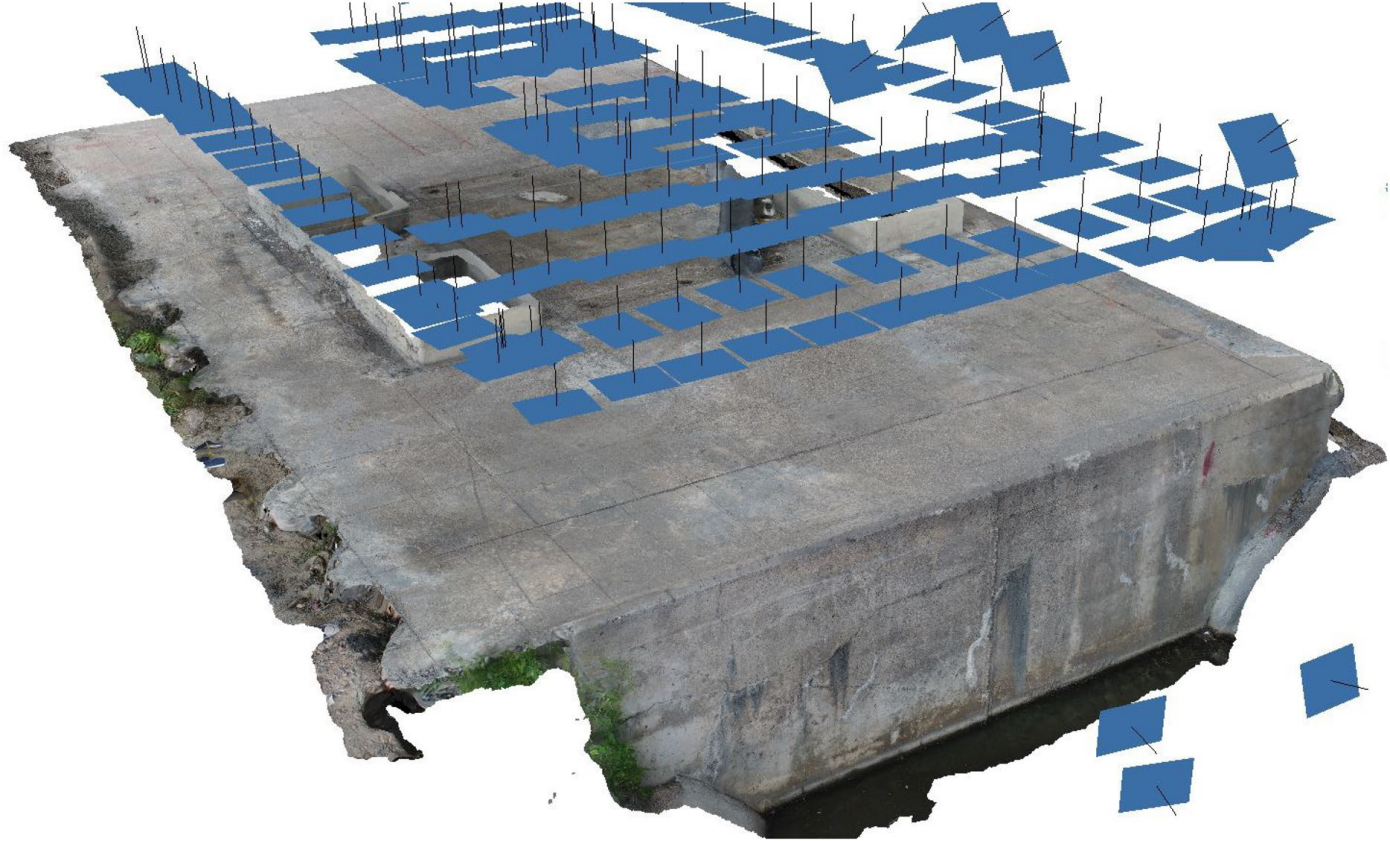
Data collection’s pre-planned path using a sweeping strategy.

When compared to other techniques, using a sweeping strategy has a number of benefits. First of all, it provides extensive coverage of the entire building, including hard-to-reach areas, surpassing the limitations of human inspection methods. Secondly, it enables the creation of detailed 3D models of the building’s surface by utilizing high-resolution photographs of its exterior. Figure 2 demonstrates the sweeping strategy. In order to ensure thorough coverage of the examined area, the unmanned aerial vehicle (UAV) uses a zig–zag pattern to travel through and survey the selected areas at a predetermined height above ground level. The UAVs were programmed to autonomously capture pictures every 2–3 s, ensuring a minimum 50% overlap between consecutive shots. The data obtained from the UAVs was subsequently utilized for 3D modeling, mosaicking, and dataset creation for training purposes. This method has many advantages over conventional techniques and can be a useful instrument for evaluating the state of damages in buildings.

### 3D mosaicking

For this paper, the 3D modeling process was performed using Agisoft Metashape software^26^, a program specifically designed for image-based 3D reconstruction. Using an automated feature extraction approach, this technique chooses outstanding and distinctive characteristics from an image. The dataset’s chosen features are those that stand out from nearby patterns while also matching and grouping comparable patterns. These characteristics could be local picture patches with particular qualities, edges, blobs, or corners. In the Agisoft system, the Scale Invariant Feature Transform (SIFT) function is employed during the feature extraction stage^27^. Following the feature detection, descriptor vectors that depict each feature’s appearance are created using feature descriptors. Following a putative relationship being established between these feature descriptors using feature matching algorithms, robust matching techniques are used to filter the correspondences. In Agisoft, features are matched using a brute-force search technique, and fundamental matrices are matched using the reliable Random Sample Consensus (RANSAC) algorithm^27^.

The function used in the preceding procedure creates tracks by combining images from the full dataset. These tracks are then used to initialize the optimizer through triangulation. Triangulation, in this context, refers to the process of estimating the sparse point cloud and camera poses by analyzing overlapping triplets of images. This initial estimation is then refined using a technique known as Bundle Adjustment (BA)^27^. The BA algorithm is used to iteratively adjust the positions of the 3D coordinates and camera poses to minimize the sum of distances between the reprojections of the reconstructed 3D points through the estimated cameras and 2D interest point coordinates. This approach helps to ensure that the reconstructed 3D points are as close as possible to their corresponding 2D interest points. It thus improves the accuracy of the overall estimation process. It should be noted that this method is commonly used in computer vision and photogrammetry applications and has been shown to produce reliable results in a variety of contexts^28^.

**Figure 3:**
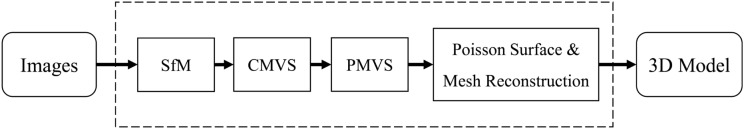
System overview of an image-based 3D modeling technique.

The technique of Structure From Motion (SFM)^27,29–31^ is employed to construct a sparse 3D model, which can subsequently be refined and enhanced to produce a more compact and detailed point cloud model. This process is facilitated by a computational algorithm known as Patch-based Multi-view Stereo (PMVS)^32^ and Poisson Surface Reconstruction (PSR)^33^, which leverages a set of images and camera parameters from SFM to reconstruct a 3D model that is progressively densified from sparse to concentrated. Following the generation of the dense point cloud, a mesh is built to enable the mapping of image texture onto the triangle mesh, producing a completely textured 3D model. Figure 3 illustrates the system overview of an image-based 3D modeling technique.

To put it simply, SFM, PMVS, and PSR are techniques that make it possible to record and interpret visual data from various angles to produce a 3D model of an object or scene. By recognizing and following features throughout a series of photos, the SFM approach creates a foundation for estimating camera postures and the 3D structure of the scene. As a result, a sparse point cloud is created, which represents the scene’s basic geometric structure but lacks the texture and richness of a more sophisticated model. To overcome this limitation, The sparse point cloud is refined and densified into a more concentrated representation using PMVS to improve the SFM-derived model. This is accomplished by creating the relevant depth maps for each pixel in the image and doing numerous analyses on the images and camera settings. These depth maps are then fused together to generate a dense point cloud that captures the detailed structure of the scene. Finally, a mesh is constructed from the dense point cloud to create a fully-textured 3D model using PSR, which can be rendered and manipulated in a variety of ways. The resulting model provides a rich visual representation of the scene.

In order to generate a mosaic in Agisoft, a mosaicking plan is first chosen. The frontal planes of the footings were chosen as the foundation for the mosaic in this study. The last stage of the mosaicking procedure entails a number of discrete phases, such as picking a compositing surface, selecting the pixels that will be included in the final composite, and blending those pixels in order to reduce the visibility of seams, blur, and ghosting. Many commercial stitching software packages have traditionally included these algorithms to produce a final composite. However, Agisoft is capable of synthesizing mosaic image data effortlessly and conveniently through the camera calibration process, utilizing the camera positions on the 3D model directly.

### Crack detection

**Figure 4:**
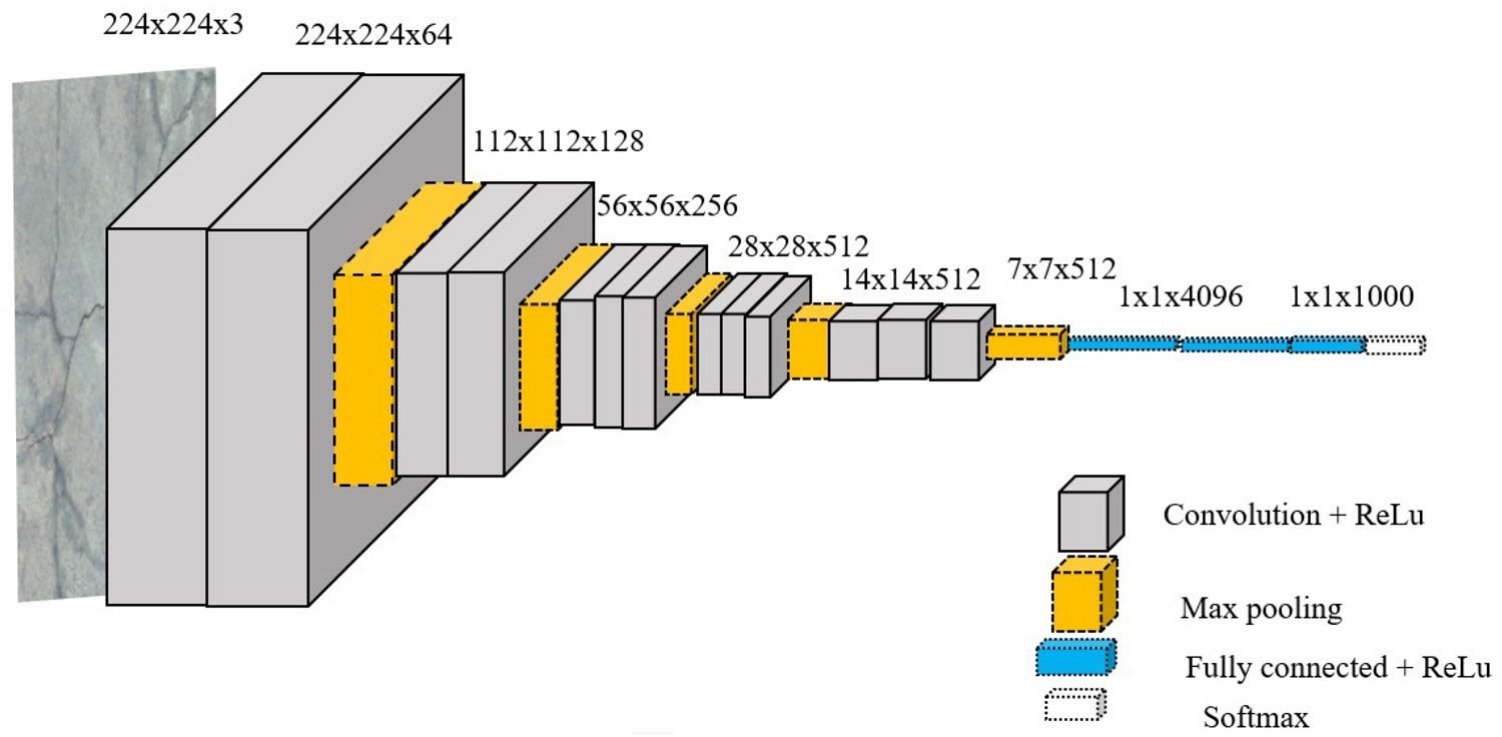
Architecture of VGG16.

In this study, the VGG16^16^ network, as illustrated in Fig. 4, was used for automatic crack detection in concrete structures, considering its capability to extract features automatically, unlike the traditional handcrafted methods. The default input size for the network is 224 $\times$ 224$\times$ 3 pixels. As illustrated in Fig. 4, input data is passed through several layers of the architecture and is generalized with a spatial size reduction of 1 $\times$ 1 $\times$ 4096 at the fully connected layer. Finally, the output is predicted as being either a crack or non-crack patch at the SoftMax layer.

The three primary responsibilities of a CNN that you mentioned are:

- Input images: The CNN receives its input as raw images. Before being fed into the network, these are often preprocessed by resizing and normalizing them.
- Deep feature extractor: The center of a CNN is the feature extractor. Through automated learning, it serves to extract useful information from the input images. This is achieved through convolutional layers, which apply filters to the input image to extract features.
- Classifier: Based on the features that the feature extractor gathered, the classifier categorizes the images into several groups. The SoftMax^34^ classifier is used to distinguish between photos with cracks and those without them.

**Figure 5:**
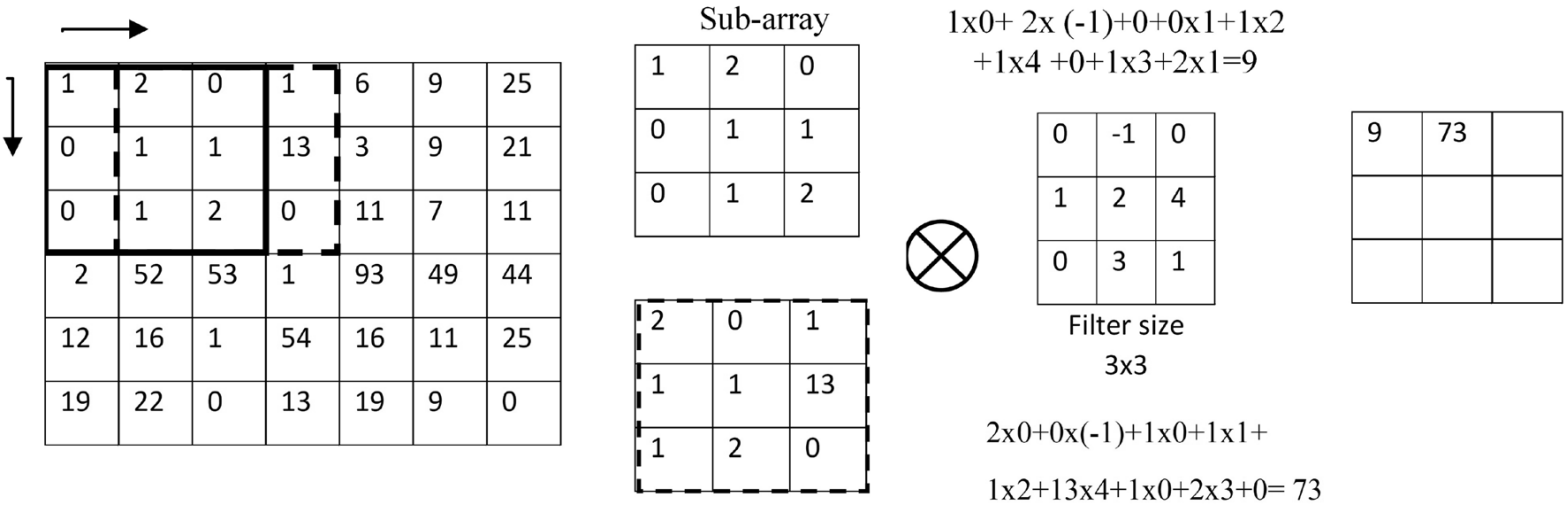
Convolution operation.

Figure 5 presents the three primary functions of the convolutional layer. The first function of the convolutional layer involves the dot product, an element-wise multiplication operation between a kernel and a sub-array of the input image. It is also referred to as a filter matrix or a receptive field. The filter size is often less than the input image’s input array size, and the filter matrix’s initial weights are created at random. Note that each operation’s sub-array of input images and the filter size are identical. In addition, it is worth noting that different filter sizes can be assigned in each convolutional layer.

The second function of the convolutional layer consists of multiplying and then combining the output values of the dot product operation. In this step, the multiplied values are added to create a scalar value for a single output feature map element. Finally, an output feature map is obtained from the output values from the second function.

**Figure 6:**
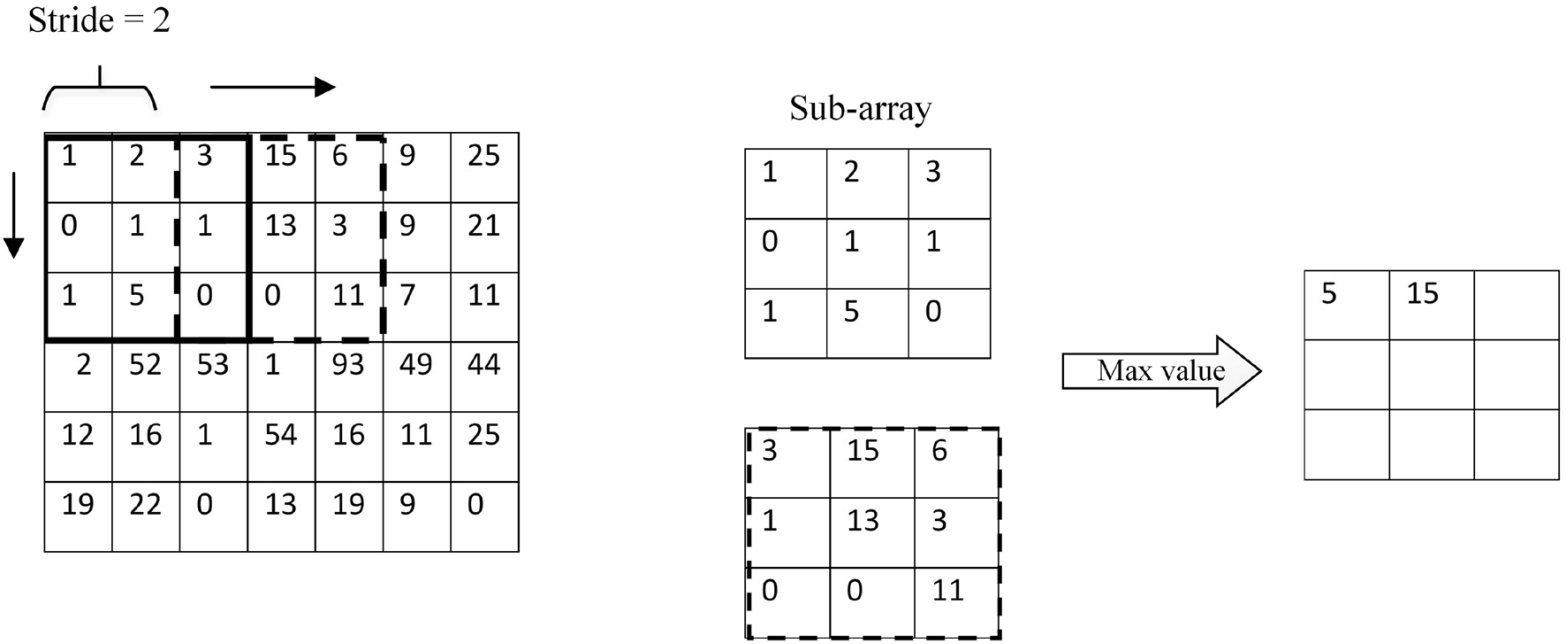
Max-pooling operation.

The CNN’s pooling layer reduces the image dimensions by reducing the number of pixels in the output it received from the previous convolutional layer. A non-linear downsampling method decreases the input pictures’ spatial dimensions throughout this procedure. This procedure uses a non-linear downsampling technique to shrink the input pictures’ spatial dimensions. The maximum value of the input image’s sub-array is chosen by the max-pooling layer, as shown in Fig. 6. Scherer et al.^35^ posit that Max-pooling outperforms the performance of the subsampling processes. Therefore, the pooling layer in the current study uses max-pooling.

The activation layer of CNNs often includes a non-linear function like $y=\;\tan h(x)$. In this study, however, the Rectified Linear Unit (ReLU) function^36^ is applied in lieu of standard $y=\;\tan h(x)$. Krizhevsky et al.^14^ demonstrated that training a CNN with the ReLU activation function can result in six times faster processing compared to conventional approaches like $y=\;\tan h(x)$. Equation 1 demonstrates the mathematical formula of the ReLU activation function $y(X)\;=\;max\;\{0,\;x\}$. It generates an output that is exclusively non-negative (zero or greater).

Overfitting remains one of the principal obstacles facing machine learning, which occurs when the data learned from training samples are effective, yet it fails to generalize for validation and testing data. Dropout layers are used to avoid overfitting. In the dropout layers, certain neurons are disconnected at random with an orderly dropout rate.

One of the most significant challenges in machine learning is overfitting, which arises when the algorithm learns well from training data but fails to generalize for validation and testing data. To mitigate overfitting, dropout layers are incorporated into the model. In dropout layers, particular neurons are randomly disconnected with a pre-specified dropout rate^37^.

During classification, a SoftMax function^34^ is used in the CNN’s final layer, as explained in Eq. 1. Given a training set $s=\;\{a^{(i)},\;b^{(i)}\}$, consisting of $n$ image patches, where $a^{(i)}$ represents the $i\textrm{th}$ image patch and *b*(*i*) denotes its corresponding class label, zero for a non-crack patch, and 1 for a crack patch, and the output of unit $j$ in the last layer of $a^{(i)}$ is denoted by $k_j^{i}$. The probability $P$ of the label *b*(*i*) of $a^{(i)}$ can be determined using Eq. 1 below, in which the scores are transformed into probabilities for the output classes:

1 $$\begin{aligned} P\left( b^{(i)}=j\backslash k_j^{i}\right) \;=\;\frac{e^{k_j^{(i)}}}{\displaystyle \underset{m}{\sum e^{k_m^{(i)}}}}\; \end{aligned}$$

The following is the SoftMax loss function $L$ for Eq. 1:

2 $$\begin{aligned} L=\;{\textstyle \frac{1}{n}}\left[\overset{n}{\underset{i=1}{\sum {\textstyle \sum _{j=1}^{i}}}}1\{b^{(i)}=j) \log \frac{e^{k_j^{(i)}}}{e^{k_m^{(i)}}}\right] \end{aligned}$$

### Data augmentation

As previously addressed, overfitting remains one of the principal obstacles facing machine learning, particularly with deep CNNs. Data augmentation is a technique by which one may artificially increase a given dataset with the aid of label-preserving transformation. Data augmentation remains the customary and undemanding method to reduce overfitting. The data augmentation consists of transforming the original image into horizontal and vertical reflections with the rotation angle $\theta$, the equations for the new coordinates of a pixel ($x', y'$) as follows:

3 $$\begin{aligned} \begin{array} {l}x'=\;x_{o}cos\theta \;+\;y_{o}sin\theta \\ y'=y_{o}cos\theta \;-\;x_{o}sin\theta \\ \end{array} \end{aligned}$$

where $x_{o}$ is the distance from the origin in the horizontal axis, $y_{o}$ is the distance from the origin in the vertical axis. The Gaussian function is given as follows, where $\delta$ is the standard deviation.

4 $$\begin{aligned} G(x_{o}, y_{o})=\;\frac{1}{\sqrt{2\pi \delta ^{2}}}e^{-\frac{x_{o}^{2} + y_{o}^{2}}{2^{\delta ^{2}}}} \end{aligned}$$

Gaussian noise (*N*) is added to the original images ($I_{o}$) with $\mu =0\;$ and $\delta ^{2}\;<\;[0.1,\;0.9]$ where the final noise-ridden image equation is as shown as in Eq. 5:

5 $$\begin{aligned} I'=\;I_{o}+\;N \end{aligned}$$

To blur the image, the value of $\delta =4$ is used. Finally, the re-scaling of the image is done with the aid of Eq. 6:

6 $$\begin{aligned} \begin{array} {l}x'\;=\;\frac{x_{o}}{y_{o\;}\times y'}\\ y'=\frac{y_{o}}{x_{o}\times y'} \end{array} \end{aligned}$$

**Figure 7:**
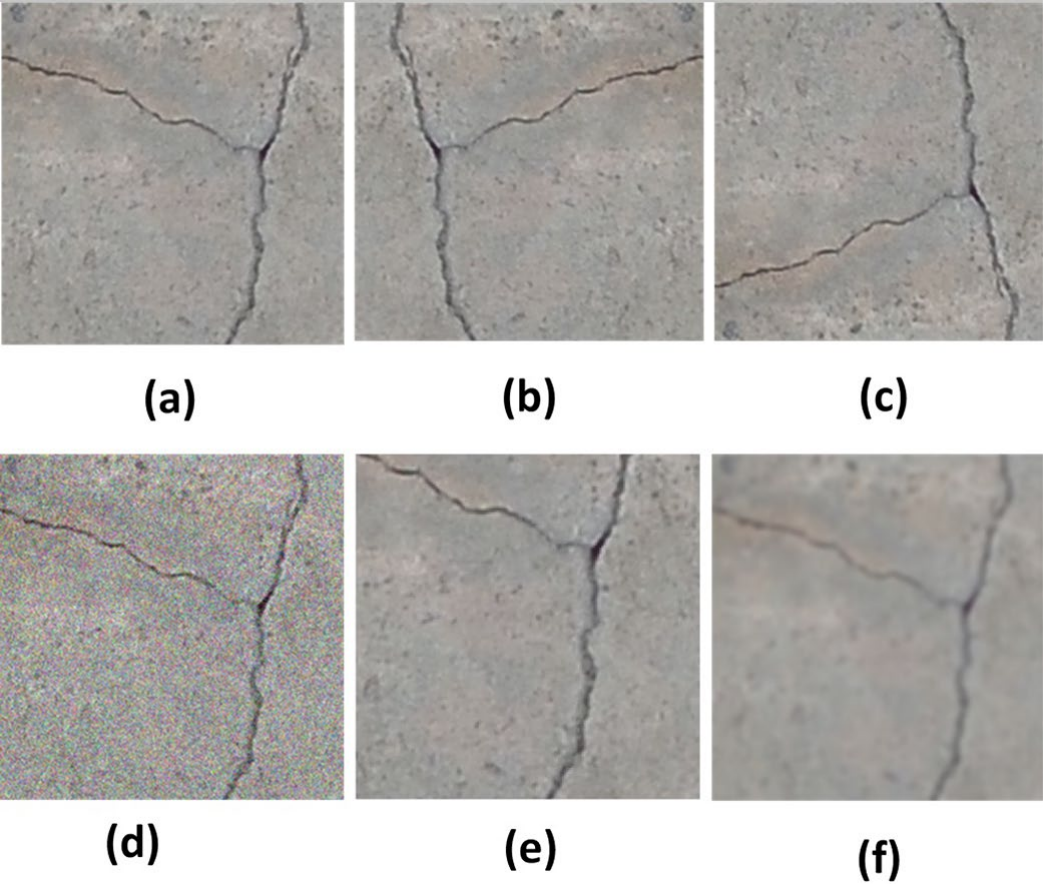
Data augmentation: (**a**) Original image, (**b**) Horizontal flip, (**c**) Vertical flip, (**d**) Gaussian noise, (**e**) Scaled patch, (**f**) Blur patch.

These functions effectively increase the training dataset by five times larger than the original data set. The sample augmented images are shown in Fig. 7.

### Confusion matrix

To evaluate the effectiveness of crack detection, a set of metrics is utilized, including the confusion matrix and classification report. These metrics are derived from the values of True Positives (TP), True Negatives (TN), False Positives (FP), and False Negatives (FN), which are determined based on the classification results of identified pixels. TP represents the number of pixels that are correctly identified as cracks (positives), while TN denotes the number of pixels that are accurately identified as non-cracks (negatives). Conversely, FP signifies the number of pixels that are erroneously identified as cracks, whereas FN represents the number of pixels that are incorrectly identified as non-cracks.

The precision Eq. 7 , recall Eq. 8, and F1 score Eq. 9 are key measures used to evaluate the performance of the crack detection algorithm. Precision is defined as the ratio of the number of TPs to the total number of pixels identified as cracks (both TPs and FPs). Recall, on the other hand, is the ratio of the number of TPs to the total number of actual cracks in the image (both TPs and FNs). F1 score is the harmonic mean of precision and recall, providing a balanced evaluation of the algorithm’s performance.

7 $$\begin{aligned} Precision\;score\;= & {} \;\frac{TP}{TP+FP} \end{aligned}$$

8 $$\begin{aligned} Recall\;score\;= & {} \;\frac{TP}{TP+FN} \end{aligned}$$

9 $$\begin{aligned} F1\;score\;= & {} \;\frac{2 \cdot Precision \cdot Recall}{Precision+Recall} \end{aligned}$$

## Experiments and results

### Dataset

**Figure 8:**
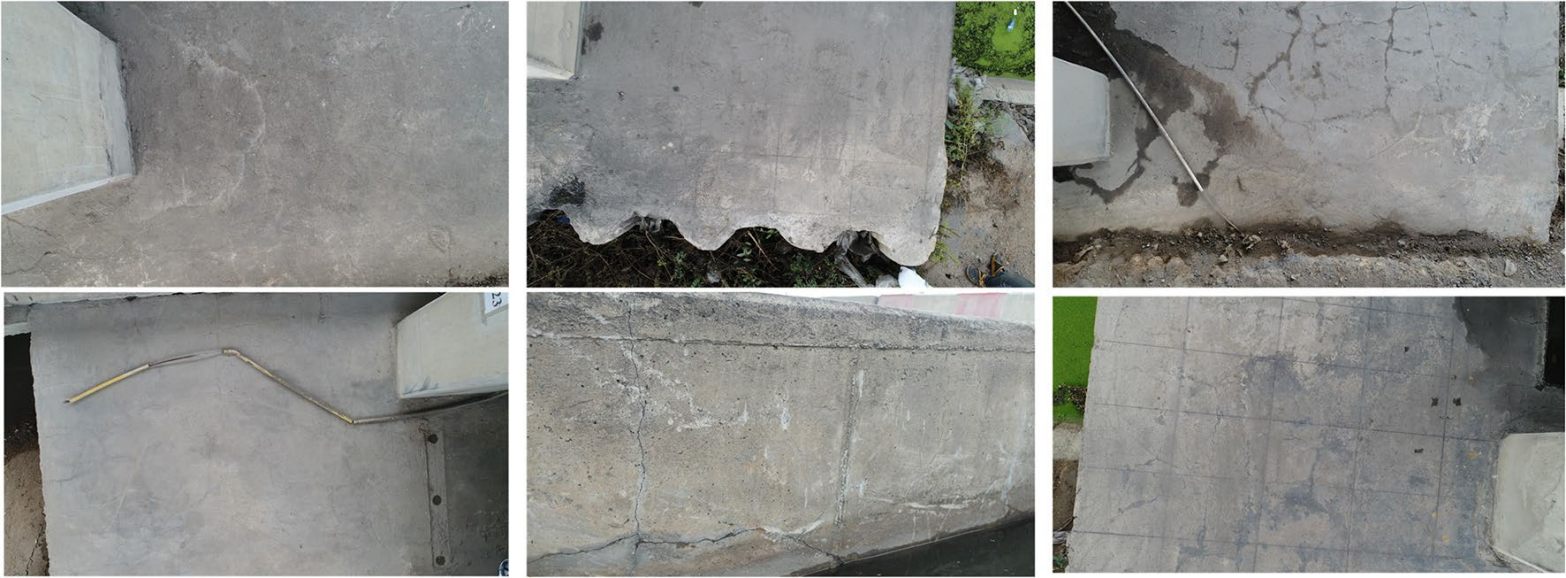
Sample image acquired using the drone.

**Figure 9:**
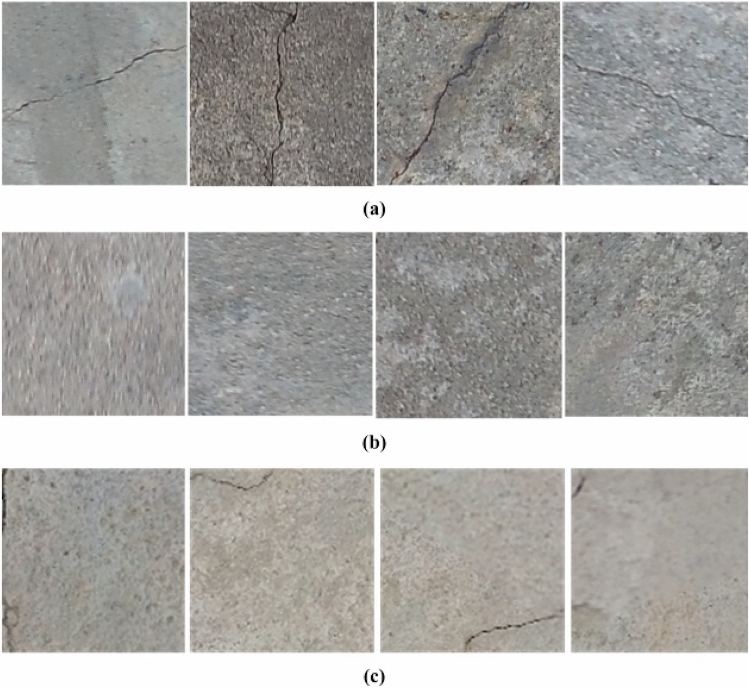
(**a**) Crack patch examples, (**b**) Non-crack patch examples, (**c**) Disregarded patches which are not included in training.

UAV image samples are displayed in Fig. 8. Following the collection of these images by the UAV, the image set was converted into patches of 224 $\times$ 224 pixels. These patches were then used to create a training dataset for the VGG16 network. A total of 11,000 patches were prepared and manually labeled into crack and non-crack categories (according to Table 1), with label 0 assigned to images with crack and label 1 assigned to non-crack images. Figure 9 displays some examples of crack and non-crack patches.

An overview of the concrete crack patch datasets is presented in Table 1. The training dataset encompassed 8000 original image patches, comprising 4000 crack-containing and 4000 non-crack-containing images. To enhance the training data’s diversity and improve model generalizability, 3000 augmented images were generated, including 1500 augmented crack images and 1500 augmented non-crack images. This augmentation process resulted in a total training dataset of 11,000 images, with approximately 1500 augmented images generated for each class. The final dataset was meticulously divided into 80% training and 20% validation subsets. The training set comprised 6400 original images and 2400 augmented images, totaling 8800 images. The validation set, on the other hand, consisted of 1600 original images and 600 augmented images, amounting to 2200 images.

**Table 1:** Summary of concrete crack patch datasets.

Dataset	No. of image patches	Crack	Non-crack	Training	Validation
Original images	8000	4000	4000	6400 (80%)	1600 (20%)
Augmented images	3000	1500	1500	2400 (80%)	600 (20%)

For training purposes, solely patch images belonging to concrete structures were selected. Patches containing surrounding objects were removed. Additionally, patches with cracks in their respective corners were strictly disregarded as these images reduce in size as they pass through multiple CNN layers and, therefore, become superfluous. Furthermore, said images may hinder the success of the classifier as it may not have the necessary features of a “crack”. Examples of such disregarded images are displayed in Fig. 9c.

### Image-based mosaicking

**Figure 10:**
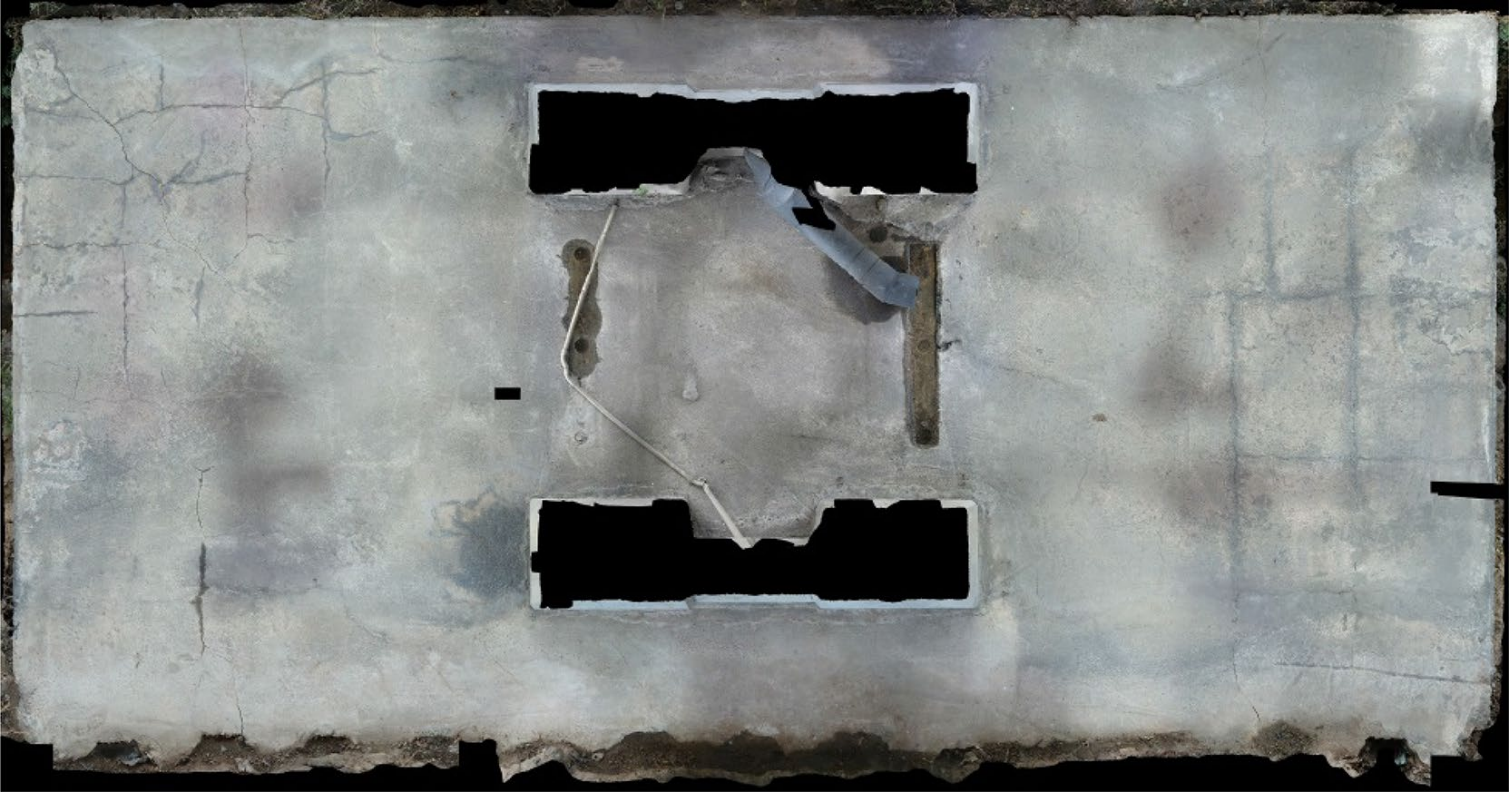
Mosaic image obtained from the input images.

A technique for 3D reconstruction using images, called Agisoft, is predominantly used for its ability to transform a set of 2D images onto a 3D point cloud of any given scene or object that it was tasked to, creating a mosaic image. The end result of the previously discussed patches are displayed in Fig. 10’s mosaic image, and reconstruction parameters are presented in Table 2.

**Table 2:** Reconstruction parameters.

Parameter name	Value
Sparse point cloud	355,955 points
RMS reprojection error	0.386051 pixels
Max reprojection error	1.17278 pixels
Dense point cloud	25,330,031 points
Surface mesh	1,688,667 faces
Vertex	848,966 vertices

### Parametric studies

In this study, experiments based on different patch sizes were performed. The patch sizes selected were 32 $\times$ 32, 64 $\times$ 64, 100 $\times$ 100 and 224 $\times$ 224. These distinct patch sizes were examined to assess the system’s precision. The results indicate that the best accuracy was achieved for the 224 $\times$ 224 patch dimension when trained and assessed with the VGG16 network. The results of the patch classification method are shown in Table 3, which is supported by a 96% accuracy rating.

**Table 3:** Different patch size experiments on validation data.

Patch size	Accuracy	Precision	Recall	F1 score
32 $\times$ 32	0.90	0.89	0.89	0.89
64 $\times$ 64	0.92	0.91	0.91	0.91
100 $\times$ 100	0.93	0.93	0.92	0.92
224 $\times$ 224	0.96	0.96	0.95	0.96

### Crack detection

To evaluate how well the suggested system works, a thorough evaluation comprising 11,000 images for training and validation was facilitated. The fully connected layers have, upon the completion of training, been re-trained according to the performance data.

Without the output layer (i.e., top layer), the pre-training model was loaded. Then, the output layer is customized to specify its classification purpose. In this study, softmax activation is used to tailor the output layer, which then categorizes each picture into crack or non-crack classes. The fine-tuning procedure is used to modify each network parameter’s value in relation to the training dataset and output layer. The end product is a model that can determine whether an image patch belongs to the ’cracked’ set. We used a batch size of 128 for all 20 epochs of training. This choice was made to balance training efficiency and model performance. Larger batch size can result in faster training, but it can also lead to overfitting, which can negatively impact the model’s performance on unseen data.

**Figure 11:**
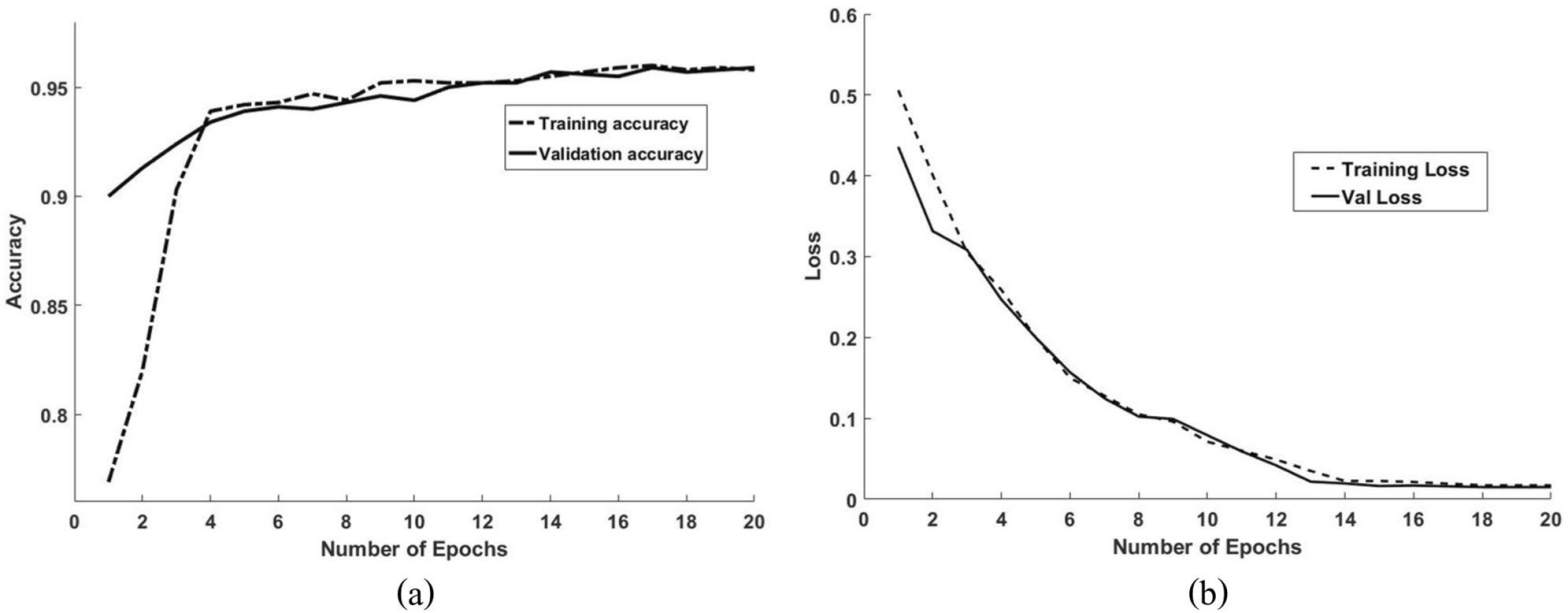
(**a**) Accuracy and (**b**) loss of the processes for training and validating VGG16 network with data augmentation.

**Figure 12:**
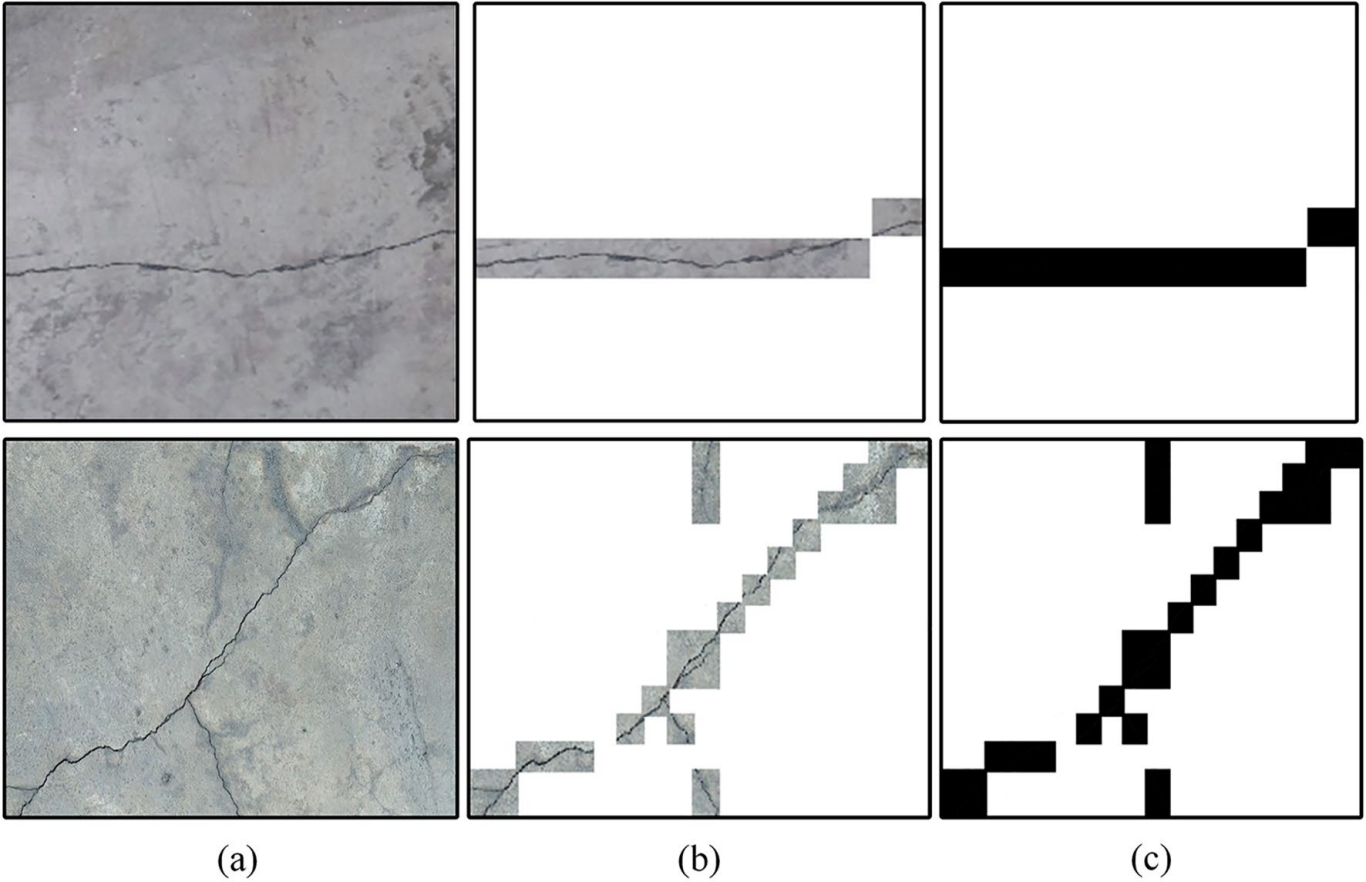
Crack Localization on mosaic small part: (**a**) Input image, (**b**) Crack localization, (**c**) Binary mask of cracks.

Accuracy of training and validation with the indicated number of epochs in Fig. 11, data augmentation was used to increase the performance of CNN and prevent overfitting. Crack localization and binary mask were obtained from the input images. The locations of the cracks in the mosaic are displayed in Fig. 12.

Finally, the proposed crack detection system was compared with the existing handcrafted features used by Prasanna et al.^11^ in which the authors implemented the STRUM classifier. In this work, the suggested method used intensity-based, gradient-based, and a mixture of scale-space characteristics as crack features. Then, these systems were collated by individual classifiers: SVM, Adaboost, and Random Forest. The features presented by Prasanna et al.^11^ indicate an accuracy score with SVM, Adaboost, and Random Forest classifiers of 87%, 85%, and 84%, respectively, as exhibited in Table 4. Additional experiments on the testing data set as shown in Table 4 concurrently. Based on its Accuracy/Precision/Recall/F1 score, the suggested system (VGG16 with Data Augmentation) performs best overall.

**Table 4:** Comparison on the testing dataset (mosaic image).

Model	Accuracy	Precision	Recall	F1 score
STRUM SVM	0.87	0.85	0.87	0.86
STRUM adaboost	0.85	0.85	0.84	0.84
STRUM random forest	0.84	0.85	0.85	0.84
VGG16 without data augmentation	0.92	0.91	0.91	0.91
VGG16 with data augmentation (proposed system)	0.95			0.95

### Data augmentation

**Figure 13:**
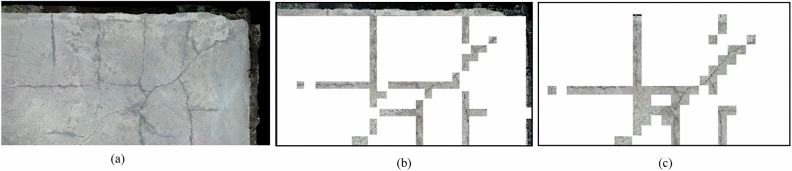
(**a**) Input image from mosaic, (**b**) VGG16 results without data augmentation, and (**c**) VGG16 results with data augmentation.

**Figure 14:**
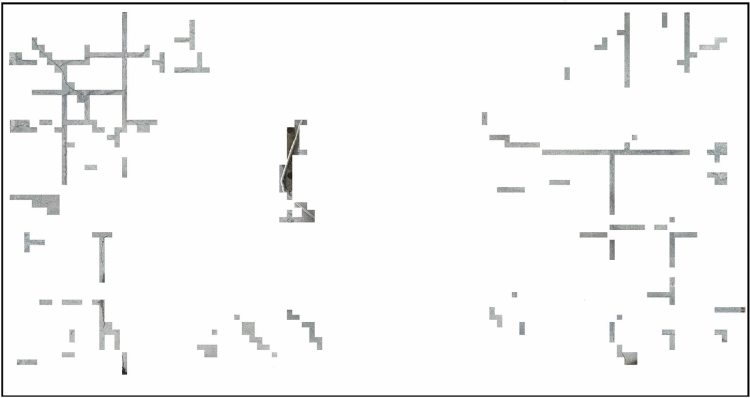
Crack Localization of full mosaic.

The results obtained on the performance of VGG16 without data augmentation exhibit an accuracy score of 92%. Figure 13a shows an example of an input image from a mosaic. Figure 13b shows an example of a non-augmented image’s outcome, which includes noise and false positives. Therefore, the data augmentation technique was utilized to create an artificial sample, by which the accuracy of the system was increased to 95%, as shown in Fig. 13c. Finally, the final results of crack localization of full mosaic, as shown in Fig. 14.

## Discussion

The results obtained from the inclusion of the VGG16 network indicate that cracks can accurately be detected and, therefore that the system may be used for automatic crack inspection. The assessed datasets were collected from different complex concrete structures using the UAV, which was then repurposed for training. For testing and visualization, an image mosaic was created from the input images. The effectiveness of the suggested strategy employing the VGG16 network shows significant improvement as compared to traditional handcrafted features. Yet, the results obtained from the VGG16 network have a chance to encounter overfitting problems, therefore, data augmentation is used as a remedy. The final crack map is obtained after processing, which includes the fusion of the VGG16 end results and thresholding. Finally, the crack map on the modeled image is smoothened and dilated to effectively visualize cracks in a large structure, which can be used for inspection purposes.

The system’s accuracy can be improved by the dataset^12,14,15^, whilst the training data’s quality can be made better by relying on the verification of experienced inspectors. Manually labeling data for training remains a time-consuming process. Therefore, one solution of many, i.e., data augmentation, is put into use. It should be mentioned that in the majority of machine learning systems, the process of producing data poses a serious challenge, and orthodox implementation of CNN networks with acceptable weights is another considerable complication. In that regard, a pre-trained network, trained on ImageNet^12^, has been retrained on the data derived in this study.

## Conclusion

In this study, a mapping system for mosaic crack detection using a VGG16 has been proposed. It is concluded from the experiments assessed herein that a VGG16 network may provide significant results in the automated finding of fractures in concrete buildings. Both validation and testing datasets have successfully used the suggested approach to recognize fractures in photos; nonetheless, it remains to be addressed that with the inclusion of higher quality and larger datasets, the performance of the system can be further improved. The results further indicate an overfitting concern for the system if a data augmentation technique is not implemented. The results also indicate a compelling increase in the accuracy of the proposed system (95%) compared to traditional handcrafted features (91%) in crack detection.

The system proposed in this study has been proven to perform better, with higher accuracy and consideration for its timeliness and efficiency, than traditional approaches in detecting cracks. A considerable factor in this performance is the CNN’s ability to automatically learn features by collecting data from images to increase the classification’s precision. The suggested system’s capacity represents a considerable advancement toward a completely autonomous examination of substantial concrete structures. The goal is to expand the dataset in the future in order to improve the suggested system’s accuracy.

The image-based crack detection system for UAV mosaic images of concrete footings shows promise. However, potential limitations include reliance on UAV imagery, affected by weather, lighting, and obstacles. Imperfections in UAV images, like blurriness or shadows, can impact performance. Future work may involve using complementary data sources and developing UAV image filtering and pre-processing algorithms. Another limitation concerns detecting cracks of various sizes and orientations using a standard VGG16 CNN. Using more sophisticated CNN architectures and exploring advanced image segmentation techniques can enhance precision in crack localization on composite images.

Besides, the evaluation based on a limited UAV image dataset prompts the need for broader research. Expanding the dataset to encompass diverse concrete footings with varying geometries, crack patterns, and environmental conditions, as well as including data from different regions with varying illumination and weather conditions, would ensure the generalizability and robustness of the proposed method. In conclusion, addressing these limitations and pursuing promising research directions can elevate the image-based crack detection system into a useful, accurate, and reliable tool for concrete footing inspection and structural health monitoring.

## Data Availability

The datasets generated during and/or analysed during the current study are available from the corresponding author upon reasonable request.
